# Minor Physical Anomalies in Adults with Autism Spectrum Disorder and Healthy Controls

**DOI:** 10.1155/2014/743482

**Published:** 2014-03-24

**Authors:** Irina Manouilenko, Jonna M. Eriksson, Mats B. Humble, Susanne Bejerot

**Affiliations:** ^1^Department of Clinical Neuroscience, Karolinska Institutet, Stockholm, Sweden; ^2^Järva Psychiatric Outpatient Clinic, Rinkebysvängen 70A, 4tr, 163 74 Spånga, Sweden; ^3^School of Health and Medical Sciences, Psychiatric Research Center, Örebro University, Örebro, Sweden

## Abstract

Minor Physical Anomalies (MPAs) are subtle abnormalities of the head, face, and limbs, without significant cosmetic or functional impact to the individual. They are assumed to represent external markers of developmental deviations during foetal life. MPAs have been suggested to indicate severity in mental illness and constitute external markers for atypical brain development. Higher frequencies of MPAs can be found in children with autism. The aims of the present study were to examine the prevalence and patterns of MPAs in adults with autism spectrum disorder (ASD) and to investigate whether MPAs are associated with symptom severity and overall functioning. Fifty adults with ASD and intelligence within the normal range and 53 healthy controls were examined with the Waldrop scale, an instrument for assessing MPAs. Face and feet were photographed enabling blinded assessment. Significant differences between the ASD and the control group were found on the MPA total scores, and also in the craniofacial region scores. Moreover, the shape of the ears was associated with autistic traits, in the ASD group. High MPA total scores were associated with poorer functioning. The findings suggest a link between MPAs, autistic traits, and level of functioning. Assessment of MPAs may assist in the diagnostic procedure of psychiatric disorders.

## 1. Introduction 

Autism spectrum disorder (ASD) is a group of neurodevelopmental disorders, characterized by atypical development, impairment in reciprocal social interaction and communication, and restricted repetitive and stereotyped patterns of behavior, interests, and activities. ASD includes autistic disorder, Asperger disorder, and pervasive developmental disorders not otherwise specified [1] and occurs in nearly 2% of the population [2]. Males are more affected than females with a sex ratio at about 4 : 1 [3]. ASD is remarkably heterogeneous and includes people with intelligence levels ranging from severe intellectual disability to very high IQ. Since no specific biological markers for ASD have been identified, the diagnosis of ASD is based on expert evaluation of cognitive, language, social, and emotional functioning along with developmental progress. In twin studies a strong evidence of genetic etiology of ASD has been shown [4–6]. Several prenatal and perinatal risk factors have also been suggested [7–10]. Furthermore, autistic traits can be measured at subthreshold level in the normal population, suggesting that the autism phenotype lies along a continuum of quantitative traits [11].

Minor Physical Anomalies (MPAs) are subtle morphological abnormalities of the craniofacial region and limbs, without significant cosmetic or functional impact to the individual. They are assumed to represent markers of deviant morphogenesis during the first or early second trimester of pregnancy and to have ectodermal embryonic origins in common with the developing brain. Genetic factors and prenatal events, such as maternal bleeding with subsequent fetal hypoxia, gestational diabetes, medication use, or toxemia, may contribute to MPAs [12, 13].

MPAs include minor malformations and phenogenetic variants that are stable over time [14].* Minor malformations* are qualitative defects of embryogenesis arising during organogenesis and are true deviations from normal.* Phenogenetic variants* are quantitative defects arising after organogenesis representing equivalents of normal anthropometric variants [15, 16].

Higher frequencies of MPAs can be found in patients with schizophrenia, bipolar disorder, ADHD, and Tourette syndrome [17–21]. In addition, MPAs are suggested to be indicators of severity of the illness [22]. Taken together, MPAs are markers for aberrant development and may be used as markers of risk for certain psychiatric disorders [23, 24].

In studies of children with ASD, MPAs are suggested to be the external markers for atypical brain development and excessive MPAs have been found when compared to neurotypically developing children [25–28]. Hitherto, only one study has examined MPAs in adults with ASD and normal intelligence. In this study interorbital and interlens distances were measured using MRI scan and showed that hypotelorism (i.e., short interorbital length) may be present in a subgroup of individuals with autism and IQ within the normal lower range [29]. However, the use of advanced methodology such as MRI for this purpose seems inappropriate for clinical practice. Accordingly, MPAs other than orbital have not been reported regarding this group. Thus it seems relevant to investigate whether MPAs contribute to the assessment of adults with ASD and normal intelligence.

The aim of the present study was (1) to investigate the prevalence, (2) to investigate the topographical pattern of MPAs in adults with ASD in comparison to neurotypical controls, and (3) to investigate whether MPAs may support diagnosis of ASD and serve as markers of impairment.

We hypothesized that (1) adults with ASD would show higher rates of MPAs than neurotypical controls; (2) the pattern of topographical distribution of MPAs would differentiate individuals with ASD from neurotypical controls; and (3) the rates of MPAs would correlate with severity of symptoms and overall functioning.

## 2. Methods

### 2.1. Participants

A total of 103 Swedish adults, including 50 adults with ASD (24 females and 26 males; mean age 30 years; range 20–47) and 53 neurotypical controls (25 females and 28 males; mean age 30.4 years; range 20–46), participated in the study (Table 1). These subjects have also been included in a study on gender coherence [30].

Inclusion criteria for all participants were age between 18 and 50 years and Caucasian descent. Exclusion criteria were any neurological or genetic syndrome, diagnosed malformations, schizophrenia spectrum disorders, intellectual disability, or having attended special education in primary or secondary school. Normal intelligence was assumed by mainstream schooling and patient records. Additional exclusion criteria for the control group were ASD in a first-degree family member, current psychiatric or personality disorder, and use of psychotropic medication.

### 2.2. Procedures and Materials

Participants were recruited between November 2006 and October 2010. Individuals with ASD and of normal intelligence were recruited through an outpatient tertiary psychiatric unit, a community-based center for adults with ASD, and also a website for people with ASD. All participants with ASD had been previously diagnosed according to the rigorous and extensive standard procedure for diagnosing ASD in Sweden at the time of the study (approximately 18 hours of assessments comprising diagnostic interviews, rating scales, neuropsychological assessments, and structured interviews with parents of the subjects) [31]. The diagnosis was further confirmed with Autism Diagnostic Observation Schedule module 4 (ADOS) [32], patient records, and a clinical interview performed by a psychiatrist experienced in ASD.

Neurotypical controls were recruited through advertisements towards a nonprofit keep-fit organization, university campuses, student residences, private companies, dentists and vaccination centers, employment agencies, and word of mouth. They were enrolled after the ASD group in order to be matched for sex and age.

Autistic traits were assessed in all participants with the Autism Spectrum Quotient (AQ): a self-report questionnaire that measures autistic traits in individuals with normal intelligence. The AQ consists of 50 items that cover abilities within social skills, communication, attention switching, attention to details, and imagination. The discriminant validity and screening properties are suggested to be satisfactory [11]. In the ASD group, the assessment of autistic traits also comprised observed traits as measured by the ADOS. The ADOS score consists of the combined communication and social interaction subscores.

Overall impairment in psychological, social, and occupational functioning was assessed with the DSM-IV Global Assessment of Functioning (GAF), shown to have good psychometric properties [1, 33]. The GAF score is a combined measure of symptom severity and level of functioning and ranges from 1 to 100. Low scores indicate severe symptoms and/or low functioning. In order to differentiate these two dimensions, severity of symptoms (GAF symptoms) and social, occupational, or school functioning (GAF-functioning) were assessed separately [34].

The study was approved by the Regional Ethical Review Board in Stockholm and written informed consents were obtained from all subjects.

### 2.3. Assessment of Minor Physical Anomalies (MPAs)

The assessment of MPAs included being photographed in a standardized manner, standing against a white wall in an examination room, which was used throughout the study. The digital photos included face close-up photos, front and in profile. Feet were photographed from beneath and from above. The participants were requested to wear a shower hat to hide the hair, but not the ears. Excessive makeup and jewelry were removed before photographing.

The Waldrop Physical Anomaly Scale [35], consisting of 16 items with total scores ranging from 0 to 25 (total MPA score), was used to assess MPAs. Head circumference was measured with a measuring tape and then categorized by reference to population based normative values (as ≥1.5 and >2 standard deviations, resp.). All other items were assessed according to descriptive anchor points, scored 0-1 or 0–2, relating to severity. Eight items (epicanthus, hypertelorism (i.e., widely spaced eyes), low-settled ears, adherent ear lobe, malformed ears, relative toe lengths, partial syndactylia, and sandal gap between first and second toe) were assessed from photographs of face (front and in profile) and feet by two psychiatrists (Irina Manouilenko, Mats B. Humble), independently and blinded to diagnosis. If their ratings on any item differed, they reached a consensus after discussion. MPAs of mouth and hands and the item “fine electric hair” were examined by two unblinded assessors (Susanne Bejerot, Jonna M. Eriksson).

According to the Waldrop Physical Anomaly Scale, the MPAs are divided into six subscales that reflect anatomic body areas: head, eyes, ears, mouth, hands, and feet. These MPAs are further classified as either minor malformation or phenogenetic variants [16] (Table 2). In addition to the total MPA score, the craniofacial index (CF-MPA) was calculated from scores of the head, eyes, ears, and mouth subscales and the periphery index (P-MPA) from scores of hands and feet subscales.

### 2.4. Statistics

Since the MPA data was not normally distributed, comparisons between the ASD group and controls were made using Mann-Whitney *U* test for continuous variables and Pearson Chi-square distribution for categorical variables. Student's *t*-test was used for analyses of normally distributed variables. Differences between groups on the MPA total scores, the subscores from the six body areas, and the CF-MPA and P-MPA were analyzed with Mann-Whitney *U* test. Missing data, assumed to be random, were present for five participants with ASD and one of the controls.

Since total MPA scores of 5 or greater have been associated with various psychiatric conditions [18, 26, 36], the participants were split into a low-MPA group, defined as having a total MPA score below 5, and the high-MPA group, defined as having a total MPA score of 5 or greater. For both groups bivariate correlations were performed to examine the relationships between MPAs, autistic traits (AQ and ADOS), and severity of symptoms (GAF-s) and overall functioning (GAF-f). Due to skewness of AQ and MPA distributions, Spearman's rank correlation test was used.

## 3. Results

Comparisons between the ASD and control groups revealed, as expected, large differences in the three symptom-related measures tested in both groups (Table 1).

**Figure 1:**
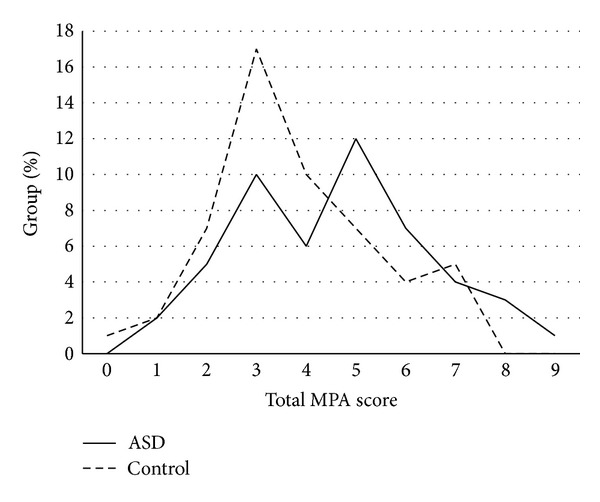
Frequency distribution of total Minor Physical Anomalies (MPA) scores in 50 individuals with autism spectrum disorder and 53 neurotypical controls.

The total MPA scores ranged from 1 to 9 (median = 5) in the ASD group and from 0 to 7 (median = 3) in the neurotypical control group; thus all participants in the ASD group displayed at least one MPA. The distribution of the total MPA score in the ASD group was bimodal; the lower mode overlapped with the distribution of the controls whereas the higher mode only appeared in the ASD group (Figure 1).

In the ASD group, the CF-MPA index, head subscale score, and MPA total score were significantly higher in comparison with the control group (Table 3). When analysed by gender, differences only reached significance for the head subscale score, which was higher in the ASD women than in control women (*U* = 191, *P* = 0.02, *r* = −0.33).

**Figure 2:**
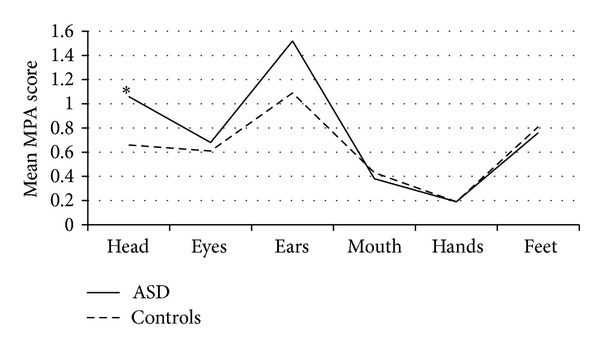
Topographical distributions of MPAs in ASD and controls. The graph shows the mean MPA score distributed on each of the six body regions in ASD and neurotypical controls, respectively.

When the sample was split into low- and high-MPA groups, 60 individuals were allocated to the low-MPA category and 43 individuals into the high-MPA group (14 women with ASD and 10 female controls and 13 men with ASD and 6 male controls). Significantly more ASD participants compared with the controls showed a high rate of MPAs (*P* = 0.014). The pattern of topographical distribution over the six body regions only showed differences between the groups in the head region (Figure 2). The single transverse palmar crease only appeared in the ASD group (*P* = 0.005); conversely the curved fifth finger was assessed as more common among the controls (*P* = 0.021), thus making the hand MPA score seemingly normal.

Testing the hypothesis that MPAs are related to functioning and autistic traits, the correlations between the MPA measures, that is, the total MPA score, CF-MPA index, and the MPA subscales, and GAF, AQ, and ADOS scores were calculated (Table 4). In the control group a pattern of correlation could only be observed between head MPA subscale and GAF-s in the high-MPA group (data not shown); thus, all other correlations presented here were derived from the ASD group alone.

In the high-MPA group, the number of MPAs correlated moderately negatively with overall functioning (GAF-f) and with severity of psychiatric symptoms (GAF-s). In the low-MPA group, moderate correlations could be observed between total MPA scores and both self-reported and observed autistic traits. In the entire ASD group, number of ear MPAs and CF-MPAs correlated weakly with the ADOS score and, negatively, with GAF-functioning. When the ASD group was analyzed for women and men separately, the above correlations could only be observed in the ASD men (Table 4). Amongst the ASD participants in the high- versus low-MPA groups no significant differences appeared in the AQ, ADOS, or GAF score. However, numerically the high-MPA group was assessed with more autistic traits according to AQ median (quartiles) scores (33 [27, 37] versus 29.0 [19, 36]) and slightly lower functioning (data not shown) compared with the low-MPA group.

## 4. Discussion

Adults with ASD and with intelligence within the normal range showed higher rates of MPAs compared with neurotypical controls. Differences between the groups were mostly found in the craniofacial region and in the hand. Higher rates of MPAs in the ASD participants were associated with somewhat lower level of functioning. Our findings are in line with earlier reports on children with ASD showing high rates of MPAs [26, 28] as well as correlations between facial phenotypes and clinical and behavioral characteristics [37]. For measuring MPAs we used the Waldrop Physical Anomaly Scale [35] with items originally developed in the midsixties for identifying children with schizophrenia. Plausibly if these children were to be assessed today, they would more likely be diagnosed with ASD.

Early in foetal morphogenesis, the development of structures in the craniofacial region occurs simultaneously with central structures of the brain implicated in the pathogenesis of, for example, ASD [38–40]. Possibly, in analogy with schizophrenia, early cerebrocraniofacial dysmorphogenesis reflects an early stage of pathogenesis that occurs before behavioral manifestations [41]. The nonspecificity of MPAs is comparable with the nonspecificity of the genetics implicated in schizophrenia, ADHD, ASD, and bipolar disorder [42] and supports the idea of biological markers transgressing the boundaries of categorical diagnoses [43]. The potential specificity of the MPAs, possibly distinguishing these disorders, has not been fully explored. Some MPAs were reported to be common in bipolar disorder [17] while they were rare amongst our participants with ASD. In order to improve the understanding of the pathophysiological mechanisms linked to MPAs, future studies should include genetic information related to these disorders.

Self-report questionnaires in addition to clinical assessment of behavioral characteristics are widely used in diagnostic procedures. However, the mentalizing deficits that characterize ASD may impair the ability for self-assessment of autistic traits, especially in those with severe ASD. Additional examination of objective morphological markers may in such cases support biological underpinnings for the dysfunction. Especially MPAs in the ear region were associated with more observable autistic traits according to the ADOS in the high-MPA group. Both the craniofacial index and the ear index were associated with higher ADOS scores and lower functioning in the entire ASD group. External ear malformations, such as low-seated ears, adherent ear lobes, and posteriorly rotated ears, have been associated with autism in several studies [12, 25, 27, 44]. Also, a possible link between external and middle ear anomalies has been proposed in children with autism [45]. Probably, the developmental anomalies of ears occur during neural tube formation in the first month of gestation—a time point when the developing brain is particularly sensitive to various teratogenic factors [46, 47]. Consequently, assessment of ear MPAs is suggested to be of interest in diagnosis and in evaluation of functional impairment in adults with ASD. However, in contrast to previous studies of children, in which minor anomalies of the ears discriminated effectively children with autism (mostly with intellectual disability) from typically developing children [13, 25, 27], this was not the case in the present study. Plausibly, the difference is due to the normal intellectual functioning of the current participants. Nevertheless, it is of interest that ASD, also within the normal intelligence range, is associated with Minor Physical Anomalies.

Notably, a substantial subgroup of our neurotypical controls exhibited MPAs, but mostly in the lower range and comparable to scores shown in other neurotypical control groups [18, 26, 28, 48, 49]. Moreover, whereas the ASD group was almost equally distributed between having high and low total MPA scores, most of the controls (70%) fell into the low-MPA group.

### 4.1. MPAs as a Method to Differentiate ASD from Neurotypical Controls

Only two out of the 16 MPAs measured with Waldrop Physical Anomaly Scale differed between ASD and controls, the minor malformations “fine electric hair” and “single palmar crease.” Although the presence of a single palmar crease is a straightforward finding, the assessment of fine electrical hair tends to be subjective and should be interpreted with caution [22, 50].

The palmar flexion creases develop during early fetal life and arise due to interaction between genetic and environmental factors, as well as movement of the developing hand in the fetus [51]. Since the flexion movements of the hands are closely associated with joint formation and muscular function, deviations in development of the palmar creases reflect either anatomical or functional alterations of the developing hand [52, 53]. In consequence, alterations may indicate intrauterine insults early in pregnancy and may be of predictive value for developmental disorders or genetic syndromes [54]. However, as alterations in palmar creases were observed in only 14% of the individuals with ASD, a single palmar crease alone is a poor predictor.

Another possibility to differentiate the groups appeared when MPAs were combined into subscales by body region. Thus the head subscale differed between the ASD group and neurotypical controls, but none of the other five body region subscales (Figure 2, Table 3). In a study on children with ASD, increased head circumference was associated with a more severe social impairment [55]. This relationship was partially supported by our findings showing a positive association between MPAs in the craniofacial region and poorer functioning (Table 4).

### 4.2. Limitations

This study has several important limitations. First, the sample was relatively small which limits the power and increases risk for type II errors. On the other hand, we did not correct *P* values for multiple comparisons, increasing the risk for type I error. Thus, when interpreting the statistical significance of the results, these two factors should be taken into consideration. Second, some of the MPAs were not blindly assessed. However, the area where we found most associations with autistic traits and functioning, that is, the ears, was blindly assessed. The assessment from photographs by blinded psychiatrists is a strength and to our knowledge not previously utilized for this kind of assessment. Earlier studies have mostly been unblinded in the sense that people with ASD have visible social impairments that may reveal their diagnosis. Also, it could be stressful for people with ASD to undress and be examined by an unfamiliar clinician, which is avoided by using photographs. Hypothetically it is conceivable that different “subtypes” composition of the ASD sample may have somewhat different MPA profiles and different course, outcome, and, respectively, global functioning. Our participants were not “subtyped” beyond being of normal intelligence, which is another caveat of this study. But given the relatively small sample size, further subtyping was considered inappropriate. Finally, in this study only Caucasians were included because hair and shape of eyes and ears often vary between different ethnic groups. Thus, the present findings cannot be generalized to other ethnic groups. Future research should preferably include larger samples and include photographs of all body regions to enable blinded assessments.

## 5. Conclusions and Clinical Implication

In this study links were shown between MPAs, autistic traits, and level of functioning in adults with ASD. MPAs of head, ears, eyes, mouths, and hands can easily be assessed in the clinician's office. Information on MPAs may provide important information for the diagnostic process. In the current study, particularly the shape and seating of the ears are suggested to be associated with autistic traits. An association between MPA scores and severity of impairments was found. Thus more MPAs may suggest need for more supportive interventions. MPAs are suggested to serve as potential biological risk markers for psychiatric disorders, and careful examination of morphological features can be helpful in the estimation of psychological, social, and occupational functioning in psychiatric patients.

## Figures and Tables

**Table 1:** Sample characteristics of the ASD group and the neurotypical controls.

	ASD (*n* = 50)	Controls (*n* = 53)
Age, years, mean (SD)	30.0 (7.3)	30.4 (7.5)
Sex, males, *n* (%)	26 (52)	28 (53)
Education, *n*		
≤9 years	8	1
≤12 years	18	7
University level	24	45
Cohabiting with partner, *n* (%)	9 (18)	26 (49)
Having children, *n* (%)	8 (16)	11 (21)
The Autism Spectrum Quotient, mean (SD)	29.4 (9.8)	11.2 (4.9)
GAF, past month, mean (SD)		
Symptoms	55.6 (6.5)	97.5 (4.4)
Functioning	55.0 (9.2)	97.6 (4.2)
ADOS, mean (SD)		
Soc-Com	8.3 (2.9)	—
Total	10.4 (3.3)	—

ASD: autism spectrum disorder; GAF: Global Assessment of Functioning; ADOS: Autism Diagnostic Observation Schedule; Soc-Com: combined social interaction-communication score.

**Table 2:** Comparison of MPA rates between the ASD and neurotypical control groups.

Minor Physical Anomalies	Score	MM/PV	ASD *n* (%)	NC *n* (%)	*χ* ^2^ (df)	*P* value
*Head *						
Head circumference		PV				
1.5–2 SD	1		23 (46)	27 (50.9)		
>2 SD	2		8 (16)	2 (3.8)		
Fine electric hair		MM			6.6 (2)	0.037*
Hair soon awry	1		12 (24)	4 (7.5)		
Hair unmanageable	2		1 (2)	—		

*Eyes *						
Epicanthus (the point of union where upper and lower lids join the nose)		PV				
Partly covered	1		8 (16)	7 (13.2)		
Deeply covered	2		1 (2)	2 (3.8)		
Intercanthal distance/hypertelorism (approximate distance between tear ducts)		PV				
Moderate	1		22 (44)	19 (35.8)		
Extensive	2		1 (2)	1 (1.9)		

*Ears *						
Seating ears—bottom of ears in line with		PV				
Area between mouth and nose	1		8 (16)	9 (17)		
Mouth (or lower)	2		—	—		
Adherent ear lobes		MM				
Lower edges of ears extend						
Moderate/Straight back toward rear of neck	1		16 (32)	14 (26.4)		
Extensive/Upward and back toward crown of head	2		18 (36)	14 (26.4)		
Asymmetrical ears	1	PV	10 (20)	3 (5.7)		
Malformed ears	1	MM	5 (10)	4 (7.5)		

*Mouth *						
High/steepled palate		PV				
Roof of mouth:						
Flat and narrow at the top	1		12 (24)	13 (24.5)		
Definitely steepled	2		—	3 (5.7)		
Furrowed tongue (one with deep ridges)	1	MM	7 (14)	4 (7.5)		

*Hands *						
Curved fifth finger:		MM			5.5 (1)	0.019^a^
Moderately curved	1		2 (4)	10 (18.9)		
Extensively curved	2		—	—		
Single transverse palmar crease	1	MM	7 (14)	—	78.0 (1)	0.005*
Fifth-finger stubbing	1	MM	1 (2)			

*Feet *						
Third toe:						
Equal in length to second	1	PV	2 (4)	—		
Definitely longer than second	2		—	—		
Partial syndactylia of second and third toes	1	MM	—	3 (5.7)		
Big gap between first and second toes	1	PV	36 (72)	40 (75.5)		

ASD: autism spectrum disorder; NC: neurotypical controls; MM: minor malformation; PV: phenogenetic variants; ^a^Higher scores in controls; level of significance *P < 0.05. Items are assessed according to descriptive anchor points (scored 0-1 or 0–2) depending on severity and “0” is defined as “no deviation”.

**Table 3:** Comparison of MPA scores by Mann-Whitney *U* test.

	ASD (*n* = 50)		Neurotypical controls (*n* = 53)		*U* (*z*)	*P *	*r*
	Mean (SD)	Median	Mean (SD)	Median			
CF-MPA	3.64 (1.97)	4	2.69 (1.51)	3	990 (−2.2)	0.025	0.22
Head	1.06 (0.89)	1	0.66 (0.67)	1	1001 (−2.3)	0.022	0.23
Eyes	0.68 (0.77)	1	0.61 (0.82)	0	1213 (−0.6)	n.s.	
Ears	1.52 (1.27)	1.5	1.09 (1.07)	1	1069 (−1.8)	n.s.	
Mouth	0.38 (0.60)	0	0.43 (0.60)	0	1255 (−0.6)	n.s.	
P-MPA	0.95 (0.69)	1	1.0 (0.65)	1	1262 (−0.5)	n.s.	
Hands	0.19 (0.43)	0	0.19 (0.40)	0	1314 (−0.1)	n.s.	
Feet	0.76 (0.52)	1	0.81 (0.48)	1	1259 (−0.5)	n.s.	
Total MPA	**4.59 (1.91)**	**5**	**3.79 (1.67)**	**3**	**1010 (−2.1)**	**0.034**	**0.21**

ASD: autism spectrum disorder; MPA: Minor Physical Anomalies; CF-MPA: craniofacial MPAs including MPAs in head, eyes, ears, and mouth; P-MPA: periphery index including MPAs in hands and feet; *U* (*z*): Mann-Whitney *U*; *r*: Pearson effect size.

**Table 4:** Correlations between MPA total, ear MPA, CF-MPA, AQ, and GAF scores for the ASD group, by MPA level (high or low) and gender.

ASD individuals	*n* (%)		Ear index (ear MPA)	Craniofacial index (CF-MPA)	Total MPA
Low-MPA (total MPA < 5)	23 (46)	AQ	0.16	0.39	0.46*
		GAF-s	−0.04	−0.04	−0.05
		GAF-f	−0.29	−0.41	−0.14
		ADOS	0.30	0.56**	0.37

High-MPA (total MPA > 5)	27 (54)	AQ	0.10	−0.20	−0.08
		GAF-s	−0.25	−0.54**	−0.57**
		GAF-f	−0.30	−0.50**	−0.52**
		ADOS	0.43*	0.30	0.25

All	50 (100)	AQ	0.23	0.20	0.23
		GAF-s	−0.06	−0.12	−0.13
		GAF-f	−0.30*	−0.36*	−0.31*
		ADOS	0.30*	0.30*	0.23

Men	26 (52)	AQ	0.42*	0.38	0.35
		GAF-s	−0.002	−0.012	−0.05
		GAF-f	−0.40	−0.48*	−0.49*
		ADOS	0.49*	0.53**	0.50**

Women	24 (48)	AQ	0.070	0.031	0.12
		GAF-s	−.052	−0.18	−0.14
		GAF-f	−0.19	−0.20	−0.093
		ADOS	0.14	0.12	0.013

ASD: autism spectrum disorder; MPA: Minor Physical Anomalies; GAF-s: Global Assessment of Functioning-symptoms; GAF-f: Global Assessment of Functioning-functioning; AQ: the Autism Spectrum Quotient; ADOS: ADOS-4 social interaction-communications score. Significances of correlations (Spearman's rank correlation test) are denoted by *P < 0.05, **P < 0.01.
